# Incidental Detection of Microfilariae in *Saguinus bicolor* and *Saguinus midas* From Central Amazon

**DOI:** 10.1111/jmp.70060

**Published:** 2026-01-14

**Authors:** Christiano T. Figueiredo, David F. Conga, Larissa Q. Pereira, Alessandra Ferreira Dales Nava, Marcelo Gordo

**Affiliations:** ^1^ Programa de Pós‐graduação Em Ciências Do Ambiente e Sustentabilidade Na Amazônia Universidade Federal Do Amazonas Manaus Amazonas Brazil; ^2^ Grupo de Medicina da Conservação e Saúde Única, Instituto de Desenvolvimento Sustentável Mamirauá Tefé Amazonas Brazil; ^3^ Instituto Federal de Educação, Ciência e Tecnologia Do Amazonas, Campus Manaus Zona Leste Manaus Amazonas Brazil; ^4^ Laboratório de Ecologia de Doenças Transmissíveis Na Amazônia, Instituto Leônidas e Maria Deane—Fiocruz Amazônia Manaus Amazonas Brazil; ^5^ Laboratório de Biologia da Conservação, Instituto de Ciências Biológicas Universidade Federal Do Amazonas Manaus Amazonas Brazil

**Keywords:** Brazilian Amazon, callitrichidae, neotropical primates, onchocercidae

## Abstract

Callitrichid primates *Saguinus bicolor* and *Saguinus midas* from urban accidents in peri‐urban forests from Central Amazon were necropsied. Analysis of thoracic and peritoneal fluid showed that 56.5% (13/23) of *S. bicolor* individuals and 13.3% (4/30) of *S. midas* individuals had the presence of *Dipetalonema* and *Mansonella* (*Tetrapetalonema*) microfilariae.

## 1 Introduction

The filarial worms that naturally infect Primates Platyrrhini (PP) are restricted to the genus *Dipetalonema*, parasitizing the thoracic and abdominal cavities, and the genus *Mansonella* (*Tetrapetalonema*), parasitizing subcutaneous tissue [1, 2]. The larval forms of these nematodes, called microfilariae, are found in the bloodstream of their hosts and exhibit morphometric and morphological characteristics used as taxonomic keys, such as the presence of a sheath, cephalic space, body size, and tail shape [3, 4]. In human filariasis, these characteristics of microfilariae are widely described and facilitate the diagnosis of diseases related to each species [5]. In veterinary medicine, there is a lack of detailed information on the morphology and morphometry of microfilariae in PP, which is useful for rapid diagnosis of filarial infection in both captive animals and environmental monitoring in wild animals.

## 2 Materials and Methods

**FIGURE 1:**
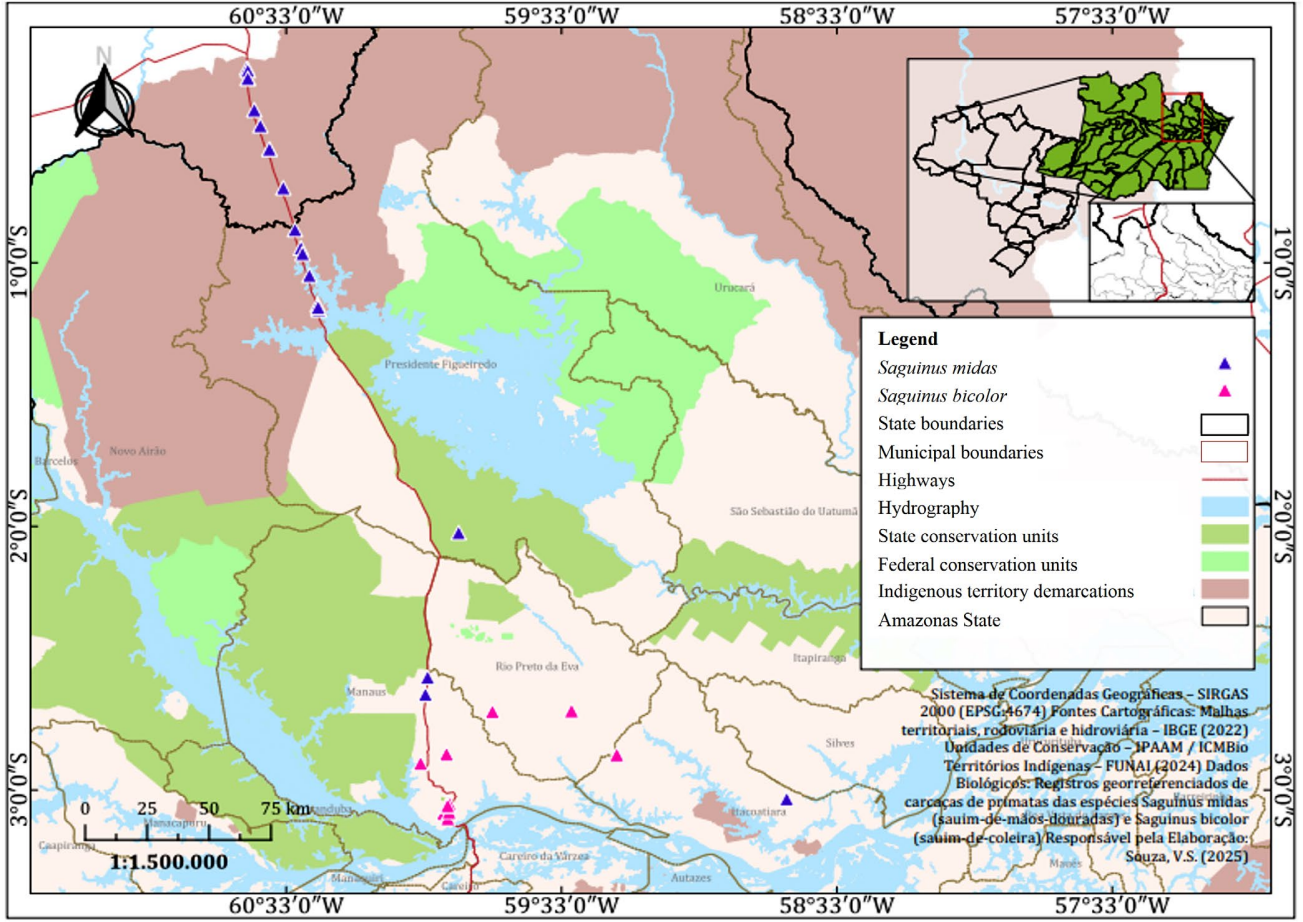
Map showing sample collection of *Saguinus midas* and *Saguinus bicolor* in the Waimiri Atroari Indigenous Territory and the municipalities of Presidente Figueiredo, Manaus, Rio Preto da Eva, and Itacoatiara (Amazonas State, Brazil).

Necropsies were performed on 53 adult callitrichids (30 *Saguinus midas* and 23 *S. bicolor* ) from accidents (electroshock and roadkill) from Central Amazon: Waimiri Atroari Indigenous Territory and the municipalities of Presidente Figueiredo (BR174 and AM 240), Manaus (*Universidad Federal de Amazonas*, *Complejo Acariquara*, *Distrito Industrial*, *Reserva Adolpho Ducke*), Rio Preto da Eva (AM 010) and Itacoatiara (Figure 1) and kept frozen in the collection of the *Projeto Sauim‐de‐Coleira* (2010–2022). During the necropsies, smears were made on slides of fluid samples from the thoracic and abdominal cavities and stained with Panótico Rápido. Morphological and morphometric characteristics of microfilariae were analyzed and taxonomically classified using the microfilariae identification keys in PP [3], all measurements are given in micrometers and average values followed by standard deviation. Adult nematodes recovered from the abdominal cavity were stored in 70°GL ethanol and clarified in Amann's lactophenol for morphological studies.

## 3 Results

**FIGURE 2:**
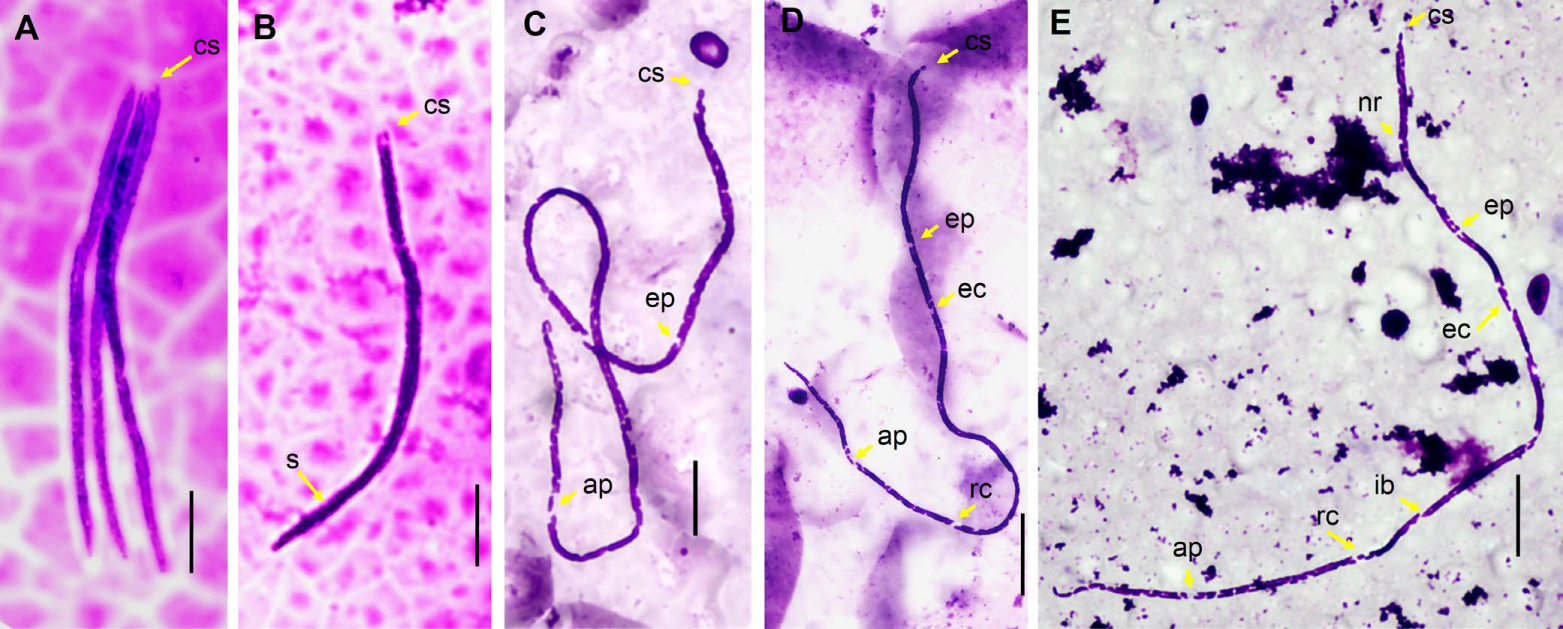
(A, B). Microfilaria of *D. gracile*, showed short and robust body with short cephalic space (cs) and sheath (s). bar: 20 μm. (C, D and E). Microfilaria of *Mansonella* (*T*.) *mariae*, body of the microfilariae with compact column of the nuclei purple in color, with exception of areas without nuclei (yellow arrow), cephalic space (cs), nerve ring (nr), excretore pore (ep), excretore cells (ec), inner body (ib), rectal cells (rc), anal pore (ap), bar: 25 μm.

In total, 56.5% (13/23) individuals of *S. bicolor* and 13.3% (4/30) of *S. midas* examined were observed to have microfilariae. Based on morphology and morphometry, the microfilariae analyzed were classified into four morphotypes: The first morphotype (36 microfilariae analyzed), observed only in *S. midas* , had a short and robust body, reduced cephalic space, short conic tail, and sheath retracted to the body, a total length of 117.9 (SD 5.6) × 3.3 (SD 0.46) in width at mid‐body length (Figure 2A,B), and was classified as microfilariae of *Dipetalonema gracile*. The second morphotype (129 microfilariae analyzed) observed in *S. bicolor* and *S. midas* had a slender and elongated body with somatic nuclei arranged in two parallel rows along the body, narrowing finely towards the end of the tail, giving a vertebrate appearance with nuclei at the tip of the tail. The total length was 316.1 (SD 14.6) × 2.2 (SD 0.3) in width. In this microfilaria, it was possible to identify structures (mean to 18 specimens) such as the nerve ring (72.3, SD 9.2), excretory pore (102.8, SD 11.5), excretory cell (114.7, SD 10.7), inner body (173.9, SD 17.4), rectal cell (216.9, SD 16.4) and anal pore (263.0, SD 15.7), measurement from anterior end. This microfilariae was classified as *Mansonella* (*Tetrapetalonema*) *mariae* (Figure 2C–E).

**FIGURE 3:**
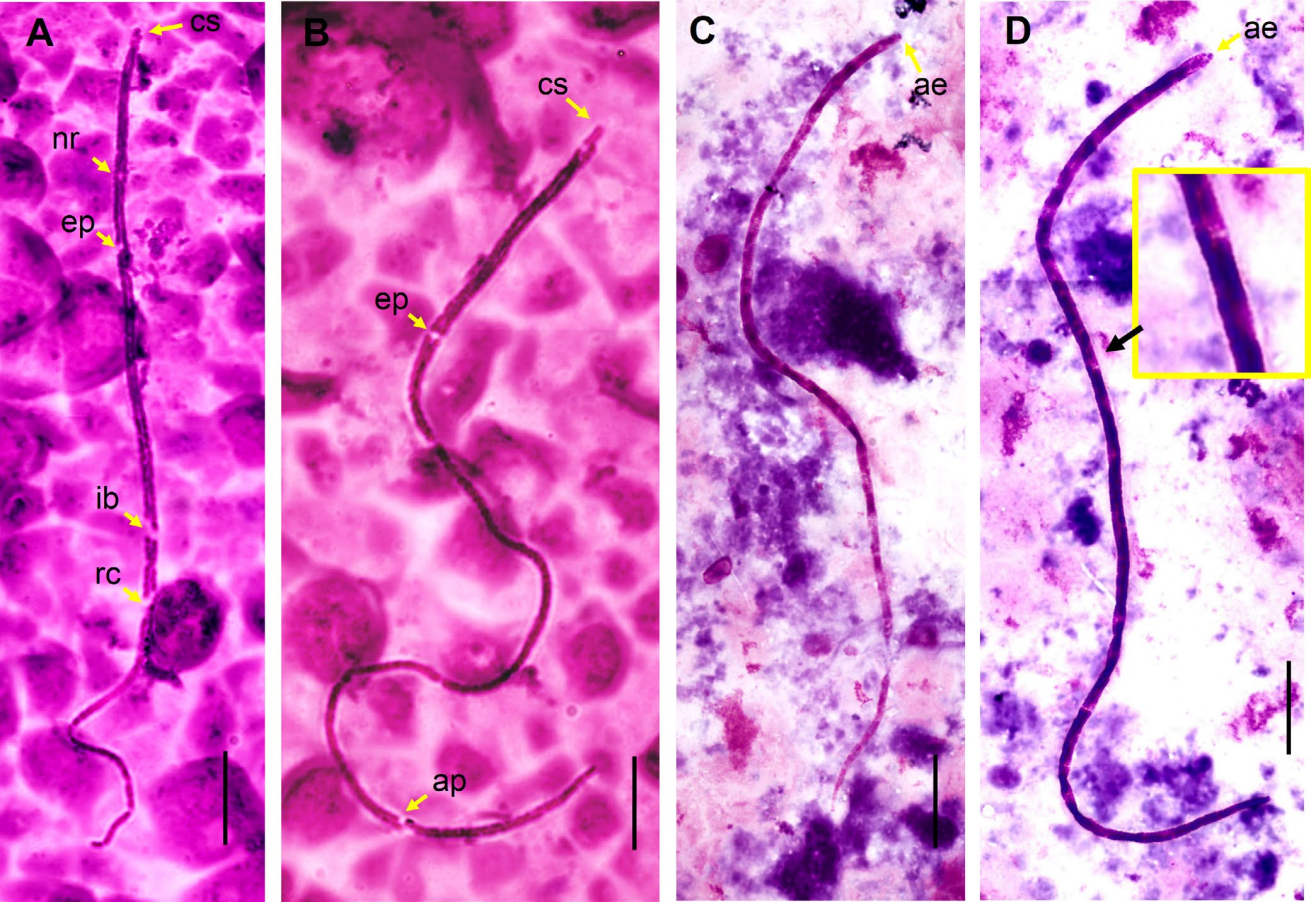
(A, B). Microfilaria of *Mansonella* (*Tetrapetalonema*). Morphotype 3, showed compact double column of the nuclei purple in color, with exception of areas without nuclei (yellow arrow), cephalic space (cs), nerve ring (nr), excretore pore (ep), inner body (ib), rectal cells (rc), anal pore (ap), bar: 25 μm. (C, D) Morphotype 4, showed rounded anterior end (ae) and body striated (black arrow).

The third morphotype (40 microfilariae analyzed) observed in *S. bicolor* and *S. midas* had a rounded anterior end, somatic nuclei arranged in two parallel rows along the body and narrowing at the tip of the tail, with nuclei at the tip of the tail, sheath absent and no striations were observed on the cuticle. The total length was 256.2 (SD 30.7) × 2.2 (SD 0.3) in width. In this microfilaria, it was possible to identify structures (mean to three specimens) such as the nerve ring (57.8, SD 4.1), excretory pore (82.1, SD 1.4), excretory cell (93.3, SD 3.4), inner body (148.0, SD 5.4), rectal cell (171.5, SD 12.3) and anal pore (217.3, SD 9.4), measurement from anterior end (Figure 3A,B). This microfilariae was classified as *Mansonella* (*Tetrapetalonema*) sp. The fourth morphotype (39 microfilariae analyzed) observed in *S. bicolor* and *S. midas* had a slender cuticle with striated markings along the body and rounded anterior end. The total length was 241.4 (SD 22.8) × 2.5 (SD 0.3) in width and it was classified within the genus *Mansonella* (*Tetrapetalonema*) sp. (Figure 3C,D). Additionally, adult filariae were recovered from the peritoneal cavity (7 females and 4 males) of *D. gracile* in an *S. midas* individual. In the *S. bicolor* individuals, no adult filariae were observed in the peritoneal‐thoracic cavities or subcutaneous tissue examined.

## 4 Discussion

Microfilariae of the genus *Dipetalonema* are characterized by the presence of a sheath, a structure observed to be highly retracted to the body of *D. gracile* microfilariae of this study, possibly due to post‐mortem changes and thawing of the host. The classification of this microfilaria is confirmed by the presence of adult specimens of *D. gracile* in the peritoneal cavity of *S. midas* . The microfilaria of *M*. (*T*.) *mariae* is one of the longest and thinnest of the genus *Mansonella*. Infection by this species was recorded in *S. bicolor* in Manaus city using molecular analysis [6]; however, details of larval morphology were not provided. This study complements this important information for the conservation of this primate, classified as critically endangered (CR) according to the IUCN [7]. For *S. midas* , infection by *M*. (*T*.) *mariae* is recorded for the first time in this study. On the other hand, the third morphotype is morphologically compatible, both in length and width, and in the presence of evident cuticular striations along the body, with the species *Mansonella* (*T*.) *marmosetae*. The fourth morphotype, classified as *Mansonella* (*Tetrapetalonema*), is morphologically compatible with the species *M*. (*T*.) *mystaxi*, both in size and width, and in the presence of caudal nuclei. However, as a limitation of this study, the presence of these two species should be confirmed by molecular analyzing and adult specimens in future research integrative on these callitrichids.

Observing adult specimens of the genus *Mansonella* at infection organ is challenging due to their minute size and the large search area in subcutaneous tissue. Microfilariae that infect humans or domestic animals were not observed in this study. However, within a One Health perspective, we highlight the relevance of monitoring filarial infections in primates inhabiting anthropized landscapes to detect the presence of *Dirofilaria immitis*, *Mansonella ozzardi*, and *Mansonella perstans* [8, 9], the latter previously reported in captive *Saguinus leocopus* [10]. On the other hand, apparently the filariae *Dipetalonema* and *Mansonella* do not have host specificity and are highly likely to be found in other platyrrhine hosts and in multispecies infections. Hyperinfections caused by adult filariae promote inflammatory reactions such as pleuritis and fibrinopurulent and fibrinous peritonitis, while microfilariae can invade various vital organs, compromising the lives of primates [6, 11, 12]. A rapid diagnosis of microfilariae using only a simple blood smear would aid in medical treatment and preventive actions, especially in captive animals and wildlife rehabilitation programs.

## Ethics Statement

This study also has a license with the *Sistema de Autorização e Informação em Biodiversidade* (SISBio), provided by the *Instituto Chico Mendes de Conservação da Biodiversidade* (ICMBio) to Marcelo Gordo (code: 10286‐7).

## Conflicts of Interest

The authors declare no conflicts of interest.

## Data Availability

The data that support the findings of this study are available from the corresponding author upon reasonable request.
